# Validating a low-cost laser speckle contrast imaging system as a quantitative tool for assessing retinal vascular function

**DOI:** 10.1038/s41598-020-64204-z

**Published:** 2020-04-28

**Authors:** Dwani D. Patel, Daniel M. Lipinski

**Affiliations:** 1 Department of Cell Biology, Neurobiology and Anatomy, Medical College of Wisconsin, Milwaukee, Wisconsin USA; 2 Department of Ophthalmology and Visual Sciences, Medical College of Wisconsin, Milwaukee, Wisconsin USA; 3 Nuffield Laboratory of Ophthalmology, University of Oxford, Oxford, UK

**Keywords:** Optical imaging, Diagnostic markers, Eye manifestations

## Abstract

The ability to monitor progression of retinal vascular diseases like diabetic retinopathy in small animal models is often complicated by their failure to develop the end-stage complications which characterize the human phenotypes in disease. Interestingly, as micro-vascular dysfunction typically precedes the onset of retinal vascular and even some neurodegenerative diseases, the ability to visualize and quantify hemodynamic changes (e.g. decreased flow or occlusion) in retinal vessels may serve as a useful diagnostic indicator of disease progression and as a therapeutic outcome measure in response to treatment. Nevertheless, the ability to precisely and accurately quantify retinal hemodynamics remains an unmet challenge in ophthalmic research. Herein we demonstrate the ability to modify a commercial fundus camera into a low-cost laser speckle contrast imaging (LSCI) system for contrast-free and non-invasive quantification of relative changes to retinal hemodynamics over a wide field-of-view in a rodent model.

## Introduction

The increasing prevalence of blinding retinal diseases with an underlying vascular etiology (e.g. age-related macular degeneration, glaucoma, and diabetic retinopathy) has made the development of technologies that enable *in vivo* assessment of vascular health a critical focal point in ophthalmic research^1^. Specifically, the ability to non-invasively and repetitively assess the onset and progression of vascular dysfunction, which precedes the appearance of sight-threatening pathologies, oftentimes by decades, would be of substantial benefit in the diagnosis and management of retinal disease^2–4^. Furthermore, evaluating vascular function in the retina may serve as a useful proxy for assessing neurological health in patients with neurodegenerative conditions, such as Alzheimer’s disease, where abnormalities in retinal blood flow have been observed prior to the onset of neurodegeneration^5,6^.

The etiology and pathophysiology of such diseases have been studied extensively using small animal models, including mice and rats, due primarily to their amenability to genetic modification, relatively short breeding time, and cost-effective housing^7^. Unfortunately, the failure of rodent models to develop the clinically detectable, proliferative vascular complications associated with end-stage retinal disease has severely limited their utility for studying disease progression long-term and for evaluating the efficacy of novel treatment options. Conversely, while large animal models such as canines and non-human primates do develop end-stage vascular complications that accurately recapitulate the human phenotype in diseases like diabetes and age-related macular degeneration, their use is severely limited by high maintenance costs and the long time frame required for pathologies to develop^7,8^. Therefore, the development of imaging techniques to accurately and reproducibly measure early, pre-symptomatic changes in vascular function would be of substantial benefit as both a diagnostic and screening tool for identifying novel biomarkers of disease progression – enabling the effectiveness of novel therapeutics to be assessed *in vivo* using small animal models that do not develop end-stage proliferative disease.

While the clinically detectable end-stage complications of proliferative retinal disease (i.e. microaneurysms, angiogenesis, hemorrhaging, and edema) may not develop in all animal models, pathologic dysregulation of retinal blood flow has often been associated with the progression of various vascular, neurodegenerative, and even psychiatric diseases^9^. Hence, the ability to detect changes in retinal hemodynamics early in disease, as an alternative to characterizing the pathologic developments observed in the late stage of disease, may identify useful biomarkers for monitoring disease progression. The emergence of advanced ocular imaging modalities such as optical coherence tomography angiography (OCT-A), doppler OCT (DOCT), and adaptive optics scanning laser ophthalmoscopy have enhanced our ability to make qualitative assessments of microvascular structure and function, but no technique is able to generate absolute quantifications of retinal hemodynamics (e.g. flow rate) over a wide field-of-view^10–12^. A promising but under-utilized modality in the context of studying retinal disease is laser speckle contrast imaging (LSCI), a non-invasive technique capable of generating wide-field, contrast-free maps of blood flow based on flow rate with high spatiotemporal resolution.

In LSCI, visualization of a diffuse surface that has been illuminated by a coherent light source (e.g. a laser) reveals a random ‘speckle pattern’ where the intensity (or brightness) of each pixel results from the coherent addition of backscattered light with different optical path lengths^13^. Movement of an object within the field-of-view induces spatial and temporal fluctuations in the speckle pattern such that the rate at which the intensity at each pixel position changes over time can be described by the decorrelation time (*τ*_*c*_) of the speckle autocorrelation function^14^. When this dynamic speckle pattern is recorded over a finite integration time (i.e. by capturing an image over a defined exposure time), the longer integration time relative to the decorrelation time results in a speckle blurring effect. The degree of blurring – referred to as speckle contrast (*K*) – can be quantified as a ratio of the standard deviation of the time integrated speckle intensities to their mean intensity (Eq. 1). Critically, *K* can be related to the speed at which an object (e.g. a blood cell) moves across a field-of-view (e.g. an image of a blood vessel in the retina) during image acquisition^15–17^. In areas of the field-of-view with faster motion, the standard deviation will decrease considerably more than the mean intensity – resulting in a reduction in speckle contrast^14^.

1 $$K=\frac{\sigma }{\langle I\rangle }$$

One major criticism of LSCI is that while speckle contrast can be used as a *relative* measure of blood flow rate, the relationship between *K* and the absolute speed of a scattering particle is non-linear^18^. Instead, the absolute speed (*ν*) of a scattering particle is inversely proportional to the decorrelation time (*τ*_*c*_) of the speckle pattern when the wavelength (λ) of the coherent light remains constant (Eq. 2)^19,20^.

2 $$v=\frac{\lambda }{2\pi {\tau }_{c}}$$

Previous mathematical models (Eq. 3) based principally on the statistical properties of speckle patterns led to the derivation of speckle correlation models relating *K* to decorrelation time (*τ*_*c*_) as a function of camera exposure time (*T*). However, in 2008, Parthasarathy *et al*. built upon the models developed by Bandyopadhyay *et al*. and Zakharov *et al*. to present a robust speckle correlation model that related *K* not only to the speed of a scattering particle through decorrelation time (*τ*_*c*_) and camera exposure time (*T*), but also normalized for the effects of speckle averaging (β), the fraction of dynamically scattered light (ρ), and the sum of experimental noise and nonzero nonergodic variance due to the spatial averaging of a random speckle pattern in the absence of flow (ϑ_n_) (Eq. 4)^13,20–25^. Importantly, using this model, Parthasarathy *et al*. were able to demonstrate linearity of relative flow estimates by an LSCI system built for cerebrovascular imaging^24^.

3 $$K(T,{\tau }_{c})={\left\{\frac{{e}^{-x}+2x-1}{2{x}^{2}}\right\}}^{\frac{1}{2}};\,x=\frac{T}{{\tau }_{c}}$$

4 $$K(T,{\tau }_{c})={\left\{\beta {\rho }^{2}\frac{{e}^{-x}+2x-1}{2{x}^{2}}+4\beta \rho (1-\rho )\frac{{e}^{-x}+x-1}{{x}^{2}}+{\upsilon }_{n}\right\}}^{\frac{1}{2}};\,x=\frac{T}{{\tau }_{c}}$$

Building upon this model, we hypothesize that it will be possible to linearly correlate relative flow measurements from a cost-effective LSCI system developed specifically for retinal imaging in small rodents to absolute measures of blood flow rate. This would enable contrast-free, wide-field maps of retinal blood flow with high spatiotemporal resolution for studying retinal hemodynamics and to potentially use as a diagnostic tool and an outcome measure of therapeutic efficacy.

However, while LSCI has been applied extensively for studying cerebral vascular flow^26^, its application for studying retinal hemodynamics is limited by several complexities. These include the need to deliver light through imperfect and aspherical optical elements (i.e. the cornea and lens) which results in spherical aberrations and imperfect focusing^27^. Moreover, the presence of micro-saccades and rapid, involuntary eye movements which may occur even under anesthesia further complicate image aquisition^28,29^. Herein, we evaluate whether modifying a commercial fundus camera into an LSCI system represents a viable and cost-effective approach to overcoming these challenges and perform LSCI in the retina of a small animal (mouse) model. We subsequently apply a multi-exposure LSCI approach and comprehensive speckle correlation model to validate whether our modified LSCI system is capable of linearly correlating measured speckle contrast values to known flow rates *in vitro* using in a model capillary tube system.

## Methods

### Animal purchase and care

Age-matched male and female C57BL/6 J mice (The Jackson Laboratory, Bar Harbor, Maine) were housed in the Biomedical Resource Center at the Medical College of Wisconsin (MCW) under a 12:12 light-dark cycle with food and water *ad libitum*. All animal protocols were reviewed and approved by the Medical College of Wisconsin Institutional Animal Care and Use Committee and conform with National Institute of Health (NIH) and Association for Research in Vision and Ophthalmology (ARVO) guidelines for the Care and Use of Laboratory Animals.

### LSCI optical design

**Figure 1:**
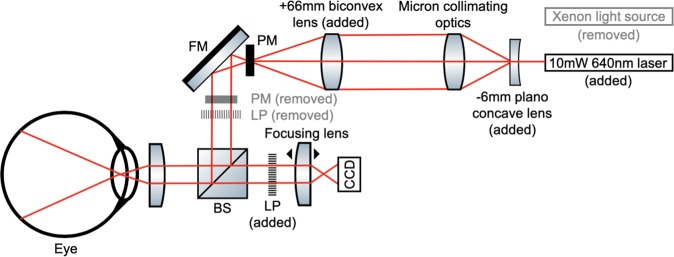
Laser Beam Schematic of Modified Micron Fundus Camera for LSCI. A xenon light source was replaced by a 10 mW 640 nm laser diode. A −6mm focal length (FL) plano-concave lens and 66.66 mm FL biconvex lens were added into the optical path to adjust the beam diameter. A pupil mask (PM) and linear polarizer (LP) were removed from the optical path, while a separate LP was introduced between the sample and the charge-coupled device (CCD). Other optical elements include a fold mirror (FM) and beam splitter (BS). Graphics used in this schematic were obtained from ComponentLibrary, a free graphics library by Alexander Franzen. Diagram not to scale.

To overcome the complexities of performing LSCI in the rodent eye, a commercial fundus camera with co-axial illumination (Micron IV, Phoenix, Pleasanton, CA) was modified by replacing the incoherent polychromatic xenon light source with a S3-Series 10 mW 640 nm coherent red laser diode (Edmund Optics, New Jersey). The laser diode was held in position using a custom 3D printed sleeve (see Supplementary Fig. S1). The 3D printed sleeve and laser diode could easily and rapidly be replaced with the incoherent polychromatic xenon light fiber and its respective fiber housing to enable bright-field fundus imaging, optic nerve alignment, and focusing between imaging sessions. A plano-concave lens with a −6mm focal length was positioned in the optical path immediately down-stream of the diode in order to expand the beam width from a point source to 22 mm without adversely affecting coherence. The expanded beam was collimated through the camera’s existing optics and finally converged to a beam width of 8 mm with an added biconvex lens with a 66.66 mm focal length (Fig. 1). A linear polarizer was introduced between the sample and the camera to exclude specular reflections. Using this modified setup, we were able to reliably illuminate an approximately 50-degree field of view, or up to 1.8 mm radially from the murine optic nerve head with the coherent laser source delivering approximately 1.5 mW power at the level of the retina. Images were captured on an 800 × 800 pixel CCD camera offering a dynamic imaging rate between 0.5 and 44 frames per second (fps) with exposure and gain adjusted using Phoenix Micron’s proprietary imaging software. The resulting spatial resolution at a single frame was ~10 μm and a temporal resolution up to 22 ms.

### *In Vivo* mouse retinal LSCI

*In vivo* retinal LSCI was performed in age-matched male and female C57BL/6 J mice under anesthesia. Anesthesia was induced using 5% isoflurane in 100% oxygen before being reduced to 2% isoflurane for maintenance. Pupils were dilated using a combination of 2.5% phenylephrine (Paragon BioTeck, Portland, OR) and 1% tropicamide (Akorn, Lake Forest, IL). Moisture of the eye was maintained during imaging with the use of Systane Ultra lubricant eye drops (Alcon Inc., Fort Worth, TX). Mice were stabilized on an imaging stage with the capability for 2 degrees of rotation and 3 degrees of translation. A xenon light source was used to find and center the optic nerve head. LSCI imaging was performed using a 10 mW 640 nm laser diode (Edmund Optics, New Jersey). For terminal imaging procedures, euthanasia was performed by intraperitoneal administration of pentobarbital (400 mg/kg).

### Controlled microcapillary flow studies using multi-exposure LSCI

Whole blood from sheep (*Ovis aries)* undergoing terminal procedures unrelated to the present study was collected in ethylenediaminetetraacetic acid (EDTA) coated specimen tubes to prevent coagulation. A glass microcapillary tube (inner diameter 1.27 mm) was connected to a 1 mL syringe with an 18-gauge needle (both Becton Dickinson, Franklin Lake, NJ). Using a syringe pump (KD Scientific, Holliston, MA), blood was forced through the microcapillary tube at various flow rates (10, 15, 20, 30, 50, 75, and 100 μL/min). For each flow rate, a stack of at least 80 images were acquired at 4.96 ms, 8.97 ms, 15.43 ms, and 27 ms exposure durations. Each frame of an individual stack was spatially processed with a window size of 5 × 5 pixels, and the spatially processed frames were averaged to generate a single noise reduced speckle contrast image representing a single flow rate at a single exposure duration. A region of interest was drawn across the perfusion area of the microcapillary tube in the speckle contrast image and the mean speckle variance (*K*^2^) calculated. *K*^2^ was plotted against exposure time for each flow rate, and data were fit to the speckle correlation model (Eq. 4) using a custom Matlab script to derive decorrelation time measurements^24^. Measured decorrelation times (*τ*_*c*_) were normalized to the baseline decorrelation time (*τ*_0_) measured at 10 μL/min (Eq. 5).

5 $${\rm{relative}}\,\tau =\frac{{\tau }_{o}}{{\tau }_{c}}$$

## Results

### Raw speckle images of the retinal vasculature can be processed to prioritize either high spatial or temporal resolution

**Figure 2:**
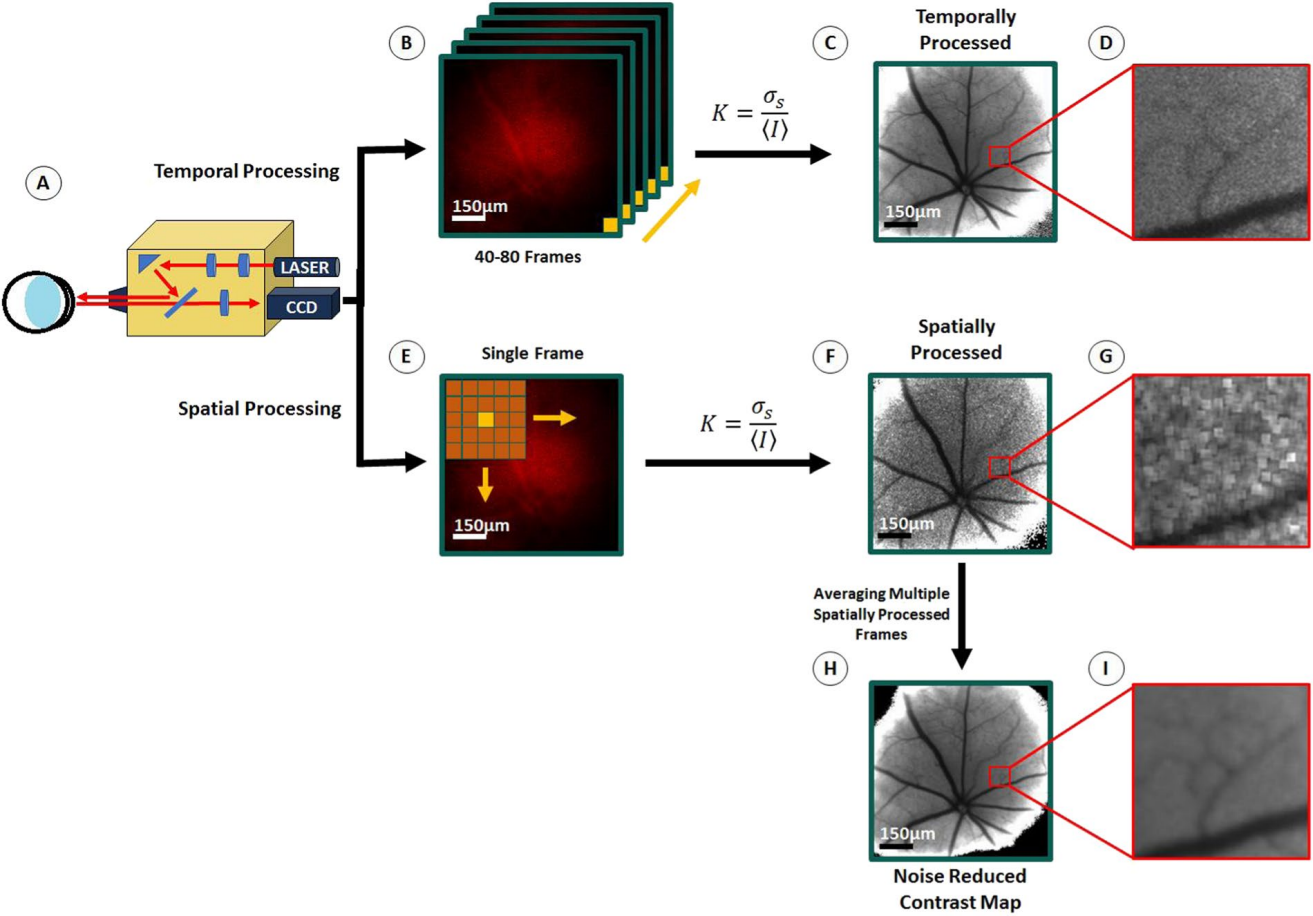
Speckle Image Processing Pipeline. A commercial fundus camera (Phoenix Micron IV) was modified by replacing a xenon light source with a 640 nm laser light source **(A)**. Acquired speckle images could be processed via a temporal processing scheme **(B)** which results in a single contrast image with high spatial resolution **(C,D)** or a spatial processing scheme **(E)** in which several spatially processed contrast images with low spatial resolution but greater temporal resolution **(F,G)** can be averaged to generate a single noise reduced contrast image with enhanced spatial resolution **(H,I)**. Scale bars: **B–F** 150 μm.

Having established that we could reliably illuminate and visualize the retina with a coherent light source using our modified system, we designed a custom laser speckle contrast analysis pipeline capable of deriving speckle contrast maps from raw speckle images using either a spatial or temporal processing scheme (Fig. 2). Prior to performing the speckle contrast analysis, image stacks were pre-processed to remove frames with substantial blurring from motion artifacts. While the mice did not experience involuntary eye movements under isoflurane anesthesia, on average, 1 out 16 frames demonstrated motion artifacts from respiratory movements. Hence, a minimum of 100 frames of data were collected to ensure at least 80 artifact-free frames were made available for subsequent contrast analysis.

In the temporal processing approach, *K* is calculated as the ratio of the standard deviation (*σ*) to the mean of speckle intensities (<*I*>) at the same pixel position over multiple consecutive frames (generally 40–80 frames) (Fig. 2B)^30^. In this approach, the temporal resolution of the resolved contrast image is compromised for greater spatial resolution (~10μm) – improving the visualization of flow in the smaller secondary and tertiary blood vessels branching of the primary retinal vessels (Fig. 2C,D).

One concern with temporal processing is that its ability to detect acute changes in vascular hemodynamics is limited by the need to average speckle intensity at each pixel location over multiple frames. An alternative approach is to process single images spatially, where *K* is calculated at each pixel location based on the statistics of speckle intensities in a small neighborhood of surrounding pixels, typically a 5 × 5 or 7 × 7 pixel grid (Fig. 2E)^17^. Unfortunately, spatial processing delivers contrast images with greater temporal resolution (up to 22 ms; limited only by the frame capture rate of the camera) at the expense of substantially reduced spatial resolution, wherein we were only able to visualize flow in large retinal arteries and veins (Fig. 2F). Encouragingly, by averaging multiple consecutive spatially processed frames (at least 80 frames) we were able to generate noise reduced contrast maps (Fig. 2H,I) with sufficient resolution to observe smaller arterioles and venules, which are indistinguishable in single spatially processed speckle images (Fig. 2G).

### Generation of a speckle contrast image of retinal vessels is dependent upon blood flow

**Figure 3:**
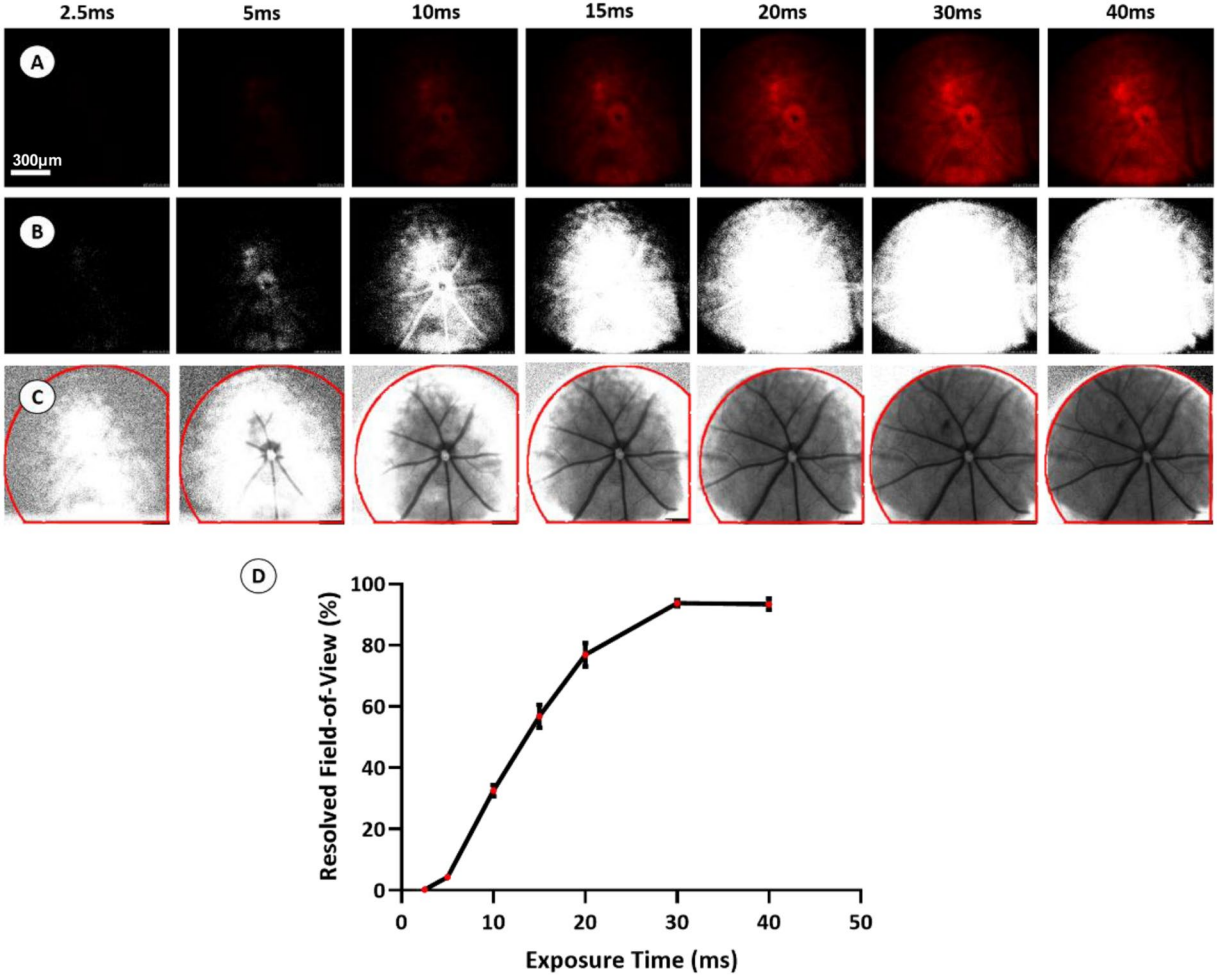
Determining the Effect of Exposure Time on Surface Illumination. *In vivo* imaging of a C57BL/6 J mouse retina was performed at multiple exposure durations to determine the effect of exposure time on surface illumination. Representative raw speckle images acquired at each exposure time are shown in (Row **A**). Each RGB raw speckle image was converted into an 8-bit image, and thresholding was applied in ImageJ to include only pixel values between 20–255 (Row **B**). Pixel values between 20–255 result in quantifiable speckle contrast intensities whereas under-sampling from insufficient laser power (pixel values <20) results in complete loss of signal (Row **C**). Consequently, the area of quantifiable speckle contrast within the total field-of-view (highlighted in red) diminishes exponentially at shorter exposure duration (**D**). Scale bar: 300 μm.

As exposure time is a fundamental variable in the speckle correlation model (Eq. 4) and shorter exposure times have been reported previously to allow for more accurate estimates of decorrelation time, we initially set out to determine the minimum exposure time required to resolve speckle contrast within retinal vessels using our modified Micron-based system. LSCI on C57BL/6 J mice at multiple exposure durations (2.5, 5, 10, 15, 20, 30, and 40 ms) under constant illumination conditions (Fig. 3A), with laser power, eye position, and all camera settings except exposure remaining unchanged between image acquisitions, revealed that the area of resolved speckle contrast diminished in a sigmoidal fashion with decreasing exposure durations (Fig. 3B–D). Based on these studies, with our current optical set-up, we estimated that an exposure of least 20 ms was required to extract meaningful speckle contrast information from more than 80% of the field-of-view. Beyond 20 ms, exposure time was not observed to substantially increase the amount of retina that could be resolved.

While the sub-optimal optical set up of our current system makes obtaining *in vivo* speckle contrast images difficult at exposure times shorter than 20 ms, the relatively uneven illumination produced by our system provided a unique opportunity to address whether stimulus uniformity is important for derivation of a speckle contrast pattern. Interestingly, we observed that so long as any given pixel was not severely under-sampled (gray value** =** <20) or over-exposed (grey value = 255) then a value for speckle contrast could be calculated. This finding is particularly important in the context of performing LSCI of the retina, where uneven illumination is highly likely owing to the necessity of providing illumination coaxially through a small aperture (i.e. the pupil) and the relatively imperfect optics of the eye, especially in models or patients with disease causing opacities (e.g. cataracts). This consideration is unique to the eye and stands in stark contrast to the situation when performing LSCI imaging in other organs, such as the brain (through the skull in mice) or skin, where achieving even illumination is relatively straightforward.

**Figure 4:**
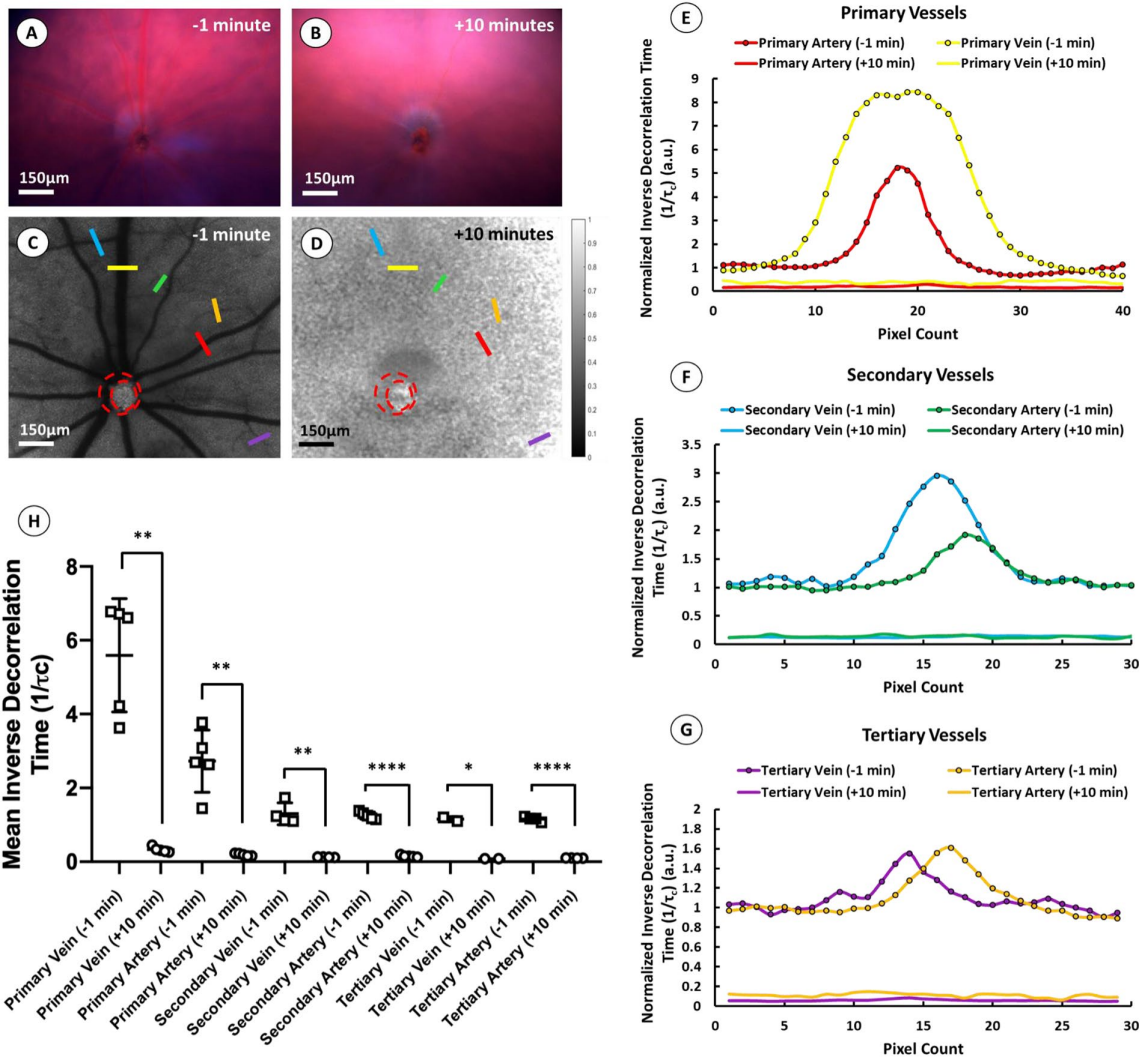
*In Vivo* Assessment of LSCI. LSCI was performed in five age-matched C57BL/6 mice (n = 5) pre- and post-euthanasia to demonstrate that speckle contrast measurements are derived from the movement of scattering particles in blood and not from the reflectance of blood vessels or absorbance of hematologic contents. Representative fundus images from a single mouse show retinal vessels 1 minute prior (**A**) and 10 minutes after euthanasia (**B**), alongside speckle contrast maps from the same mouse pre- (**C**) and post-euthanasia (**D**) (scale bar ~150μm). Cross-sectional analysis of selected primary, secondary, and tertiary vessel from the speckle contrast images shown in (**C,D**) demonstrates parabolic flow profiles prior to euthanasia which are reduced and indistinguishable from background after death (**E–G**). Paired t-tests comparing mean inverse decorrelation time (1/*τ*_*c*_) in primary, secondary, and tertiary vessels across the five mice pre- and post-euthanasia demonstrate significant reduction in 1/*τ*_*c*_ following death (**p = 0.0018, **p = 0.0029, **p = 0.0041, ****p < 0.0001, *p = 0.0289 and ****p < 0.0001, respectively) (**H**). Scale bars: **A–D** 150 μm.

While it is not possible to make absolute quantitative measurements of blood flow rate using our current system owing to the necessity of using long-duration (>20 ms) exposures, we nevertheless aimed to demonstrate that such devices can detect and quantify *relative* changes in blood flow over time. Furthermore, we intended to confirm that measured speckle contrast is derived solely from the movement of blood within the vessels and not from artifacts, such as reflectance from the vascular structures or absorbance by hematologic contents. As a consequence, we performed LSCI on C57Bl/6j mice (n** =** 5) pre- and post-euthanasia on the rationale that death is expected to cause a large and uniform change in blood flow that can be detected even when using long-duration exposures. Fundus photography performed 1-minute prior to euthanasia using a polychromatic white xenon light source demonstrated healthy retinal perfusion with blood observed in both retinal veins and arteries (Fig. 4A). Perfusion was visibly diminished 10 minutes post injection of sodium pentobarbital, although blood was still noticeably present in several retinal vessels (Fig. 4B). Noise reduced speckle contrast maps following LSCI at 25 ms exposure duration (Fig. 4C,D) demonstrate a clear reduction in blood flow following euthanasia. Interestingly, using the inverse decorrelation time (1/*τ*_*c*_) as an estimate of flow rate (Eq. 2), cross-sectional analysis of several retinal vessels demonstrates parabolic flow profiles prior to euthanasia, as would be expected if the observed speckle signal arises from laminar flow of blood (Fig. 4E–G). The speckle contrast we observed to be essentially absent in those same vessels post-euthanasia, despite blood remaining in the lumen (Fig. 4B), provides further evidence that speckle contrast in the retina is derived solely from the movement of blood, rather than from the presence of hematologic contents. Moreover, the reduction in relative flow could be accurately quantified. Paired two-tail t-tests comparing the mean inverse decorrelation time across multiple primary, secondary, and tertiary veins and arteries in five C57BL/6 J mouse retinae demonstrated a significant reduction in flow across all vessel types after death (**p** =** 0.0018, **p** =** 0.0029, **p** =** 0.0041, ****p** <** 0.0001, *p** =** 0.0289 and ****p** <** 0.0001, respectively) (Fig. 4H).

### A controlled microcapillary flow study using multi-exposure LSCI reveals a linear relationship between flow rate and decorrelation time

**Figure 5:**
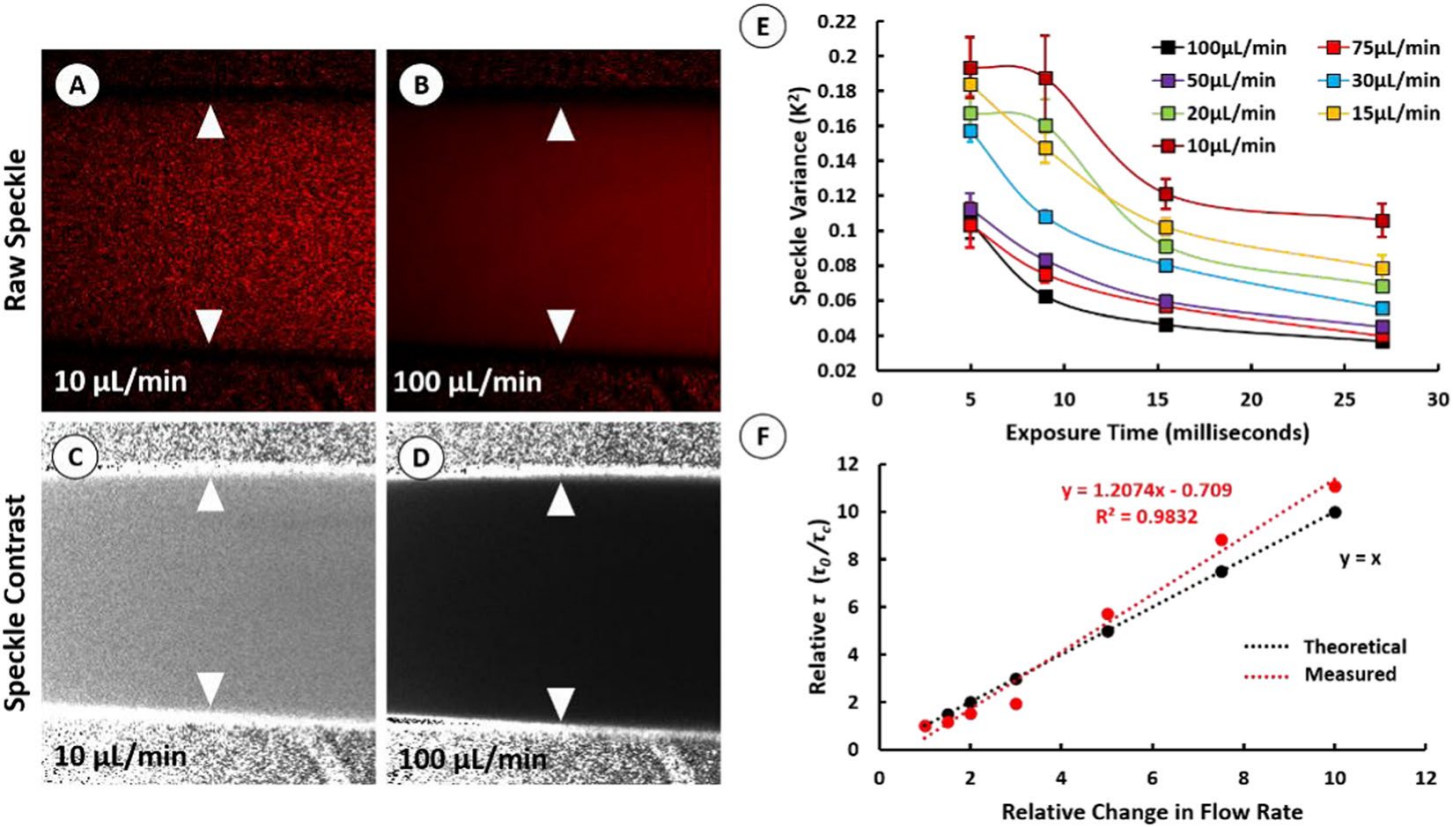
Controlled Microcapillary Flow Studies with Multi-Exposure LSCI. Individual stacks of at least 80 raw speckle images were captured at 4.96 ms, 8.97 ms, 15.43 ms, and 27 ms exposure times for various controlled flow rates (10, 15, 20, 30, 50, 75, and 100 μL/min). Each individual stack was spatially processed and averaged to generate a single noise reduced contrast map representing a single flow rate at a single exposure duration. Representative raw speckle images and noise reduced contrast maps are shown for measurements made of flow rates 10 μL/min and 100 μL/min at 4.96 ms exposure (**A–D**). The speckle variance (*K*^2^) measured from each noise reduced contrast map was plotted against exposure duration (**E**). Estimates of decorrelation time (*τ*_*c*_) for each flow rate were determined by fitting the multi-exposure dataset to a speckle correlation model as presented by Parthasarathy *et al*. (Eq. 2). Measured values of (*τ*_*c*_) were normalized to baseline decorrelation measured at 10 μL/min (*τ*_0_) and plotted against relative change in flow rate (**F**).

Controlled microcapillary flow studies were conducted to determine the linearity between LSCI based flow estimates and true values (Fig. 5). Unlike in the case of *in vivo* retinal imaging where the resolved speckle contrast image was reduced to 4.25% of the total field-of-view at 5 ms exposure, perfusion through the microcapillary tube could be fully resolved without under-sampling at exposure times as short as 4 ms – broadening the range of exposure durations in which speckle contrast could be measured (Fig. 5A–D). In this multi-exposure LSCI study, 80 frames at 24fps were captured at 4.96 ms, 8.97 ms, 15.43 ms, and 27 ms exposure durations for each of the examined flow rates (10, 15, 20, 30, 50, 75, and 100 μL/min). Following spatial processing of the raw speckle data, the mean speckle variance (*K*^2^) for each flow rate was plotted against exposure duration (Fig. 5E). The multi-exposure dataset was subsequently fit to the speckle correlation model (Eq. 4) to generate a single estimate of decorrelation time for each flow rate. The measured decorrelation time (*τ*_*c*_) for each flow rate was normalized to the baseline decorrelation time (*τ*_0_) measured at a flow rate of 10 μL/min. This multi-exposure imaging approach demonstrated a linear increase in relative *τ* with increasing flow rates (*R*^2^** =** 0.98; Fig. 5F). Importantly, while the capillary tube model presented here is a highly artificial system, these findings indicate that increasing illumination of the retina to the degree that exposure times in the low millisecond range can be used may improve sensitivity of LSCI to higher flow rates. This may further allow us to improve the linearity of measured decorrelation times with true flow rates in order to validate the approximation described in Eq. 2, and therefore allow us to accurately calibrate our LSCI system into measuring absolute flow rates.

## Discussion

A LSCI modality for quantifying rodent retinal hemodynamics in the pre-symptomatic stage of retinal vascular diseases may offer a vital non-invasive tool for detecting critical biomarkers of disease progression (i.e. changes in blood flow rate, heart rate, and vessel rigidity). However, the utility of LSCI as a diagnostic tool is highly dependent on our ability to accurately and absolutely measure true flow rates from speckle contrast measurements over time and in the same patient. While a few LSCI instruments are available for purchase and use in humans and animal models, here we examined whether it was possible to modify a commercial fundus camera found in many ophthalmic research laboratories into a LSCI system for rodent retinal imaging. Through controlled flow experiments using a microcapillary tube, we also tested the linearity between flow estimates generated by our optical design and true values. In doing so, we address the validity of LSCI as a quantitative tool for assessing retinal vascular function in rodents and highlight some of the critical design consideration that must be addressed to build a translational LSCI device.

The challenge of quantifying retinal hemodynamics with a non-invasive imaging strategy is being tackled through the application of various techniques including: laser doppler flowmetry (LDF), doppler optical coherence tomography (D-OCT), OCT-angiography, and LSCI^14^. Although the mathematical principals underlying LSCI were described several decades ago, this imaging technique remains a relatively underutilized and under-developed modality for assessing retinal hemodynamics, despite potentially offering several advantages (i.e. the ability to quantify flow in vessels running orthogonal to the illumination source over a wide-field of view) over more established technologies. Additionally, LSCI can be built with relatively cheap instrumentation while maintaining the adaptability to be combined with other imaging modalities (i.e. bright field fundoscopy, fluorescent imaging, etc.). While there are a some commercial LSCI instruments for retinal imaging, most of these systems are designed to measure *relative* changes in retinal hemodynamics. Moreover, these technologies lack the spatial resolution to adequately resolve microcapillaries where a majority of pre-symptomatic changes are expected to take place, and their image processing algorithms often use estimates of speckle contrast to minimize processing time. Hence, a robust LSCI system with capabilities of absolute quantification of retinal hemodynamics remains an unmet challenge.

While by no means a unique consideration, one of the main challenges facing development of any LSCI system for use in animals is delivering a coherent light stimulus to the retina through a narrow pupil aperture while limiting specular reflections from the cornea and lens. Several approaches to overcoming this issue have been reported previously with various degrees of success and practicality, including delivering the laser stimulus using an endoscope or ring illumination system that limits corneal refraction, or an off-axis, trans-scleral illumination technique^28,31^. The advantage of using an endoscopic approach as demonstrated by Ponticorvo *et al*. is wide-field LSCI of the rodent retinal arterioles and veins, as well as choroidal vessels in non-pigmented rat models, while correcting for corneal refraction without the use of a contact lens^28^. However, an endoscope is a single, rigid optical device that is difficult to modify, and the processed speckle contrast images are not of sufficient resolution to adequately resolve secondary and tertiary blood vessels or capillaries that branch from the primary vessels radiating from the optic nerve head. Unlike the endoscopic approach, the trans-scleral illumination technique as described by Srienc *et al*. may be able to resolve smaller retinal and choroidal vessels in pigmented and non-pigmented rodent models. However, the field of view is highly dependent on the precise position of the illuminating optical fiber on the eye’s surface – complicating the use of such a technique longitudinally where repetitively imaging the same retinal location may be critical. By contrast, the Phoenix Micron fundus camera is widely used and offers a compact and co-axial optical setup for fundus photography that can be easily modified for retinal LSCI through the incorporation of several low-cost optical elements. In our optical setup, a plano-concave lens with a −6mm focal length and biconvex lens with a focal length of 66.66 mm were added in the optical path to adjust the beam diameter, and a linear polarizer was inserted between the sample and camera to eliminate specular reflections. Moreover, the co-axial imaging setup eliminated the need to align optical elements with the camera sensor and enabled flexibility for changing light sources, allowing LSCI and bright-field fundoscopy to be performed rapidly and without manipulating the subject.

As the majority of early hemodynamic changes in diseases such as diabetic retinopathy affect the smaller capillaries and secondary and tertiary retinal blood vessels, the ability to resolve these vessels would be a significant advantage for any retinal LSCI modality^9^. As shown in Fig. 2C,D, the modified Phoenix Micron IV can clearly resolve primary retinal vessels and some secondary and tertiary blood vessels. Despite this, the spatial resolution achieved by our optical setup, or any currently available commercial LSCI imaging device, is far from that of OCT-A or D-OCT. The ability to resolve these smaller blood vessels depends greatly on the penetration depth of the laser, as well as the exposure time of the camera. LSCI with full-field illumination limits the penetration depth of the laser light to superficial vessels whereas using a line beam scanning illumination technique can improve sampling depth but at the expense of sampling rate^32^. The sensitivity to slower flow in capillaries and smaller blood vessels can also be increased at higher exposure times, but this compromises the specificity of contrast measurements in regions of high flow rate^33^. Hence, determining the optimal exposure duration to maximize signal-to-noise in rodent retinal LSCI is a critical design consideration.

Even without the spatial resolution of OCT-A to resolve smaller blood vessels, LSCI offers the ability to measure other important metrics of retinal hemodynamics which can help to characterize disease progression. For example, some clinical studies have shown significant differences in total blood flow to the retina in patients with mild to early diabetic retinopathy compared to healthy controls^34^. Hence, the ability to quantify blood flow in primary retinal vessels allows us to estimate total blood flow to retina and demand from downstream, smaller vessels. Moreover, the wide-field of view also enables us to quantify vessel diameters, flow rates, and flow profiles across multiple vessels at the same instance in the cardiac cycle. Finally, increasing the frame capture rate of the camera to satisfy the Nyquist sampling rate required for imaging the murine cardiac waveform has benefits of its own. The frame capture rate of the camera in these experiments was 24fps. This frame rate was appropriate for conducting multi-exposure studies *in vitro* where flow was continuous and not pulsatile. At this frame rate, we could also get a relative estimate of flow rate across vessel cross sections as seen in Fig. 4E–G. However, a camera with a greater frame capture rate could significantly enhance temporal resolution and reduce long averaging times to improve estimates of flow rate as well as help to extract subtle metrics such as heart rate, blood flow waveforms, pulsatility, and even vessel rigidity and compliance.

In our optical design, we were limited to imaging retinal vessels at exposure durations greater than 20 ms and imaging our microcapillary apparatus at exposure durations greater than 4 ms. With a maximum of 10 mW power output at the laser source and power attenuation throughout the optical path, we experienced under sampling at shorter exposure times and consequently our resolved field of view diminished exponentially (Fig. 3B). Interestingly, we also observed that speckle contrast computation is not highly dependent on the uniformity of illumination, such that, where pixels are not under sampled, the intensity of the resolved speckle contrast image remained relatively uniform despite high variability in the intensity of raw speckle images (Fig. 3A). While uniformity of illumination is rarely a concern when performing LSCI of other organs, such as the brain or skin, where providing flood illumination of the target tissue is relatively straightforward, it is of substantial concern when designing an LSCI instrument to image the fundus, where the illumination and imaging paths are coaxial and necessarily have to pass through a narrow pupil aperture.

Although non-uniform illumination may not be a critical concern while imaging at higher exposure times (>20 ms), maintaining adequate light intensity to avoid under-sampling at microsecond exposure durations becomes a critical design consideration – especially considering Parthasarathy *et al*. demonstrated improvements in estimates of decorrelation time while applying multi-exposure LSCI across a wide range of exposure times (50μs-80ms). In our system, we experienced underexposure while imaging the rodent retina under 20 ms exposure durations. To improve our ability to image at shorter exposure times while maintaining adequate illumination, we can incorporate a higher power laser diode to overcome power attenuation through the optical path or select a camera sensor with greater sensitivity to low light conditions. Additionally, as demonstrated by Parthasarathy *et al*., we can maintain constant camera noise and image intensity by imaging at a fixed camera exposure duration while gating the laser diode to modulate speckle exposure time^24^.

Our retinal LSCI modality also demonstrates the ability to quantify retinal blood flow in primary, secondary, and tertiary branches of the central retinal artery and vein *in vivo*. Using the inverse decorrelation time (1/ *τ*_*c*_) as a proxy for absolute speed, we observed that speckle intensity had a parabolic profile across the vessel diameter, as would be expected if contrast was generated by the laminar flow of blood within a vessel (i.e. moving faster in center of the lumen than near the vessel wall) (Fig. 4E–G)^35^. Additionally, we were able to quantify significant reductions in relative blood flow across arteries and veins following euthanasia (Fig. 4H). Together these observations support that speckle contrast is derived from the movement of blood, rather than scattering of light from the hematologic contents.

Due to the lack of a definite relationship between decorrelation time and speed, the inverse decorrelation time (1/ *τ*_*c*_) is often used as an estimate for absolute speed (Eq. 2). To determine the linearity between this proxy for absolute speed versus true values, we designed a controlled microcapillary flow system and performed LSCI over multiple flow rates. Despite our inability to image the controlled microcapillary flow apparatus at exposure durations shorter than 4 ms, we were able to fit our multi-exposure LSCI dataset to Eq. 4 and demonstrate a linear increase in relative *τ* with respect to increasing flow rates (Fig. 5E,F). In this curve-fitting exercise, the values of *β*_,_ *τ*_0,_ and ϑ_n_ were initially estimated by fitting the multi-exposure dataset at 10 μL/min flow rate to the speckle correlation model described by Eq. 4. The speckle correlation model was simplified with the assumption that the fraction of total dynamically scattered light (*ρ*) was 1 due to the absence any static scattering material between the LSCI optical setup and glass microcapillary tube. The decorrelation value, *τ*_*c*_ for each flow rate was subsequently estimated while maintaining the value of *β* constant.

While there is a direct linear relationship between our LSCI based estimates of flow rate and true values, a slope greater than 1 suggests that the error between LSCI based measurements and true values increases at higher flow rates (Fig. 5F). Increasing the laser power and improving our ability to perform LSCI at shorter exposure durations while maintaining constant illumination will critically enhance the sensitivity of our system to high flow rates and consequently enhance our ability to demonstrate a 1:1 increase in LSCI based flow estimates with true values. Nevertheless, that the current LSCI system can be used to determine relative changes to blood flow in an *in vivo* setting is highly encouraging for potential use as a diagnostic tool. We have demonstrated that modifying the Micron IV fundus camera into a retinal LSCI system has enabled non-invasive, widefield, and rapid assessments of retinal perfusion in mouse models. By achieving a 1:1 relationship between LSCI measurements and absolute values, we aim to eventually calibrate our retinal LSCI modality to derive absolute measures of particle speed.

## Conclusions

Developing a LSCI system for retinal imaging in small rodent models may offer a vital tool for studying the physiologic consequences of retinal vascular diseases on retinal hemodynamics prior to and even in the absence of clinically detectable complications which develop in late stage of disease. Consequently, a tool which can effectively study retinal hemodynamics in the early stages of disease can also help to identify novel biomarkers for monitoring disease progression and treatment efficacy in the pre-symptomatic stage of disease. Here we modify a commercial fundus camera into a relatively low-cost, co-axial, and adaptable LSCI system which can deliver temporally and spatially processed speckle contrast images with high spatial and temporal resolution, respectively. With the multi-exposure LSCI approach presented by Parthasaraty *et al*., we also demonstrate linearity in our *τ*_*c*_ based estimates of flow rate with true values in a controlled microcapillary flow apparatus. The current optical design and imaging strategy is advantageous for generating contrast-free and wide-field speckle contrast maps of primary and some secondary and tertiary retinal blood vessels without the need for costly high-power lasers or cameras with high frame capture rates. These contrast maps can also be used to determine relative changes to flow rate in primary arterioles and venules in response to blood flow altering stimuli. However, improving the linearity in relative flow estimates and resolution of capillaries and secondary and tertiary blood vessels is a critical consideration in designing a robust LSCI system for studying diseases such as diabetic retinopathy, glaucoma, and Alzheimer’s disease which affect the smaller retinal microvascular networks. In such as case, laser penetration, laser power, camera exposure, and incident light illumination become paramount design factors.

## Supplementary information

Supplementary Figure S1.

## Data Availability

The datasets generated during and/or analyzed during the current study are available from the corresponding author on reasonable request.
