# Supplementary material for: Validating a low-cost laser speckle contrast imaging system as a quantitative tool for assessing retinal vascular function
Source: Sci Rep. 2020 Apr 28;10:7177. doi: 10.1038/s41598-020-64204-z (PMC7188677; doi:10.1038/s41598-020-64204-z)
Supplement: Supplementary file 1 — Supplementary Figure S1. [file 41598_2020_64204_MOESM1_ESM.pdf]

Supplementary Information File

Manuscript Title: **Validating a low-cost laser speckle contrast imaging system as a quantitative tool for assessing retinal vascular function**

Authors: **Dwani D. Patel, Daniel M. Lipinski**

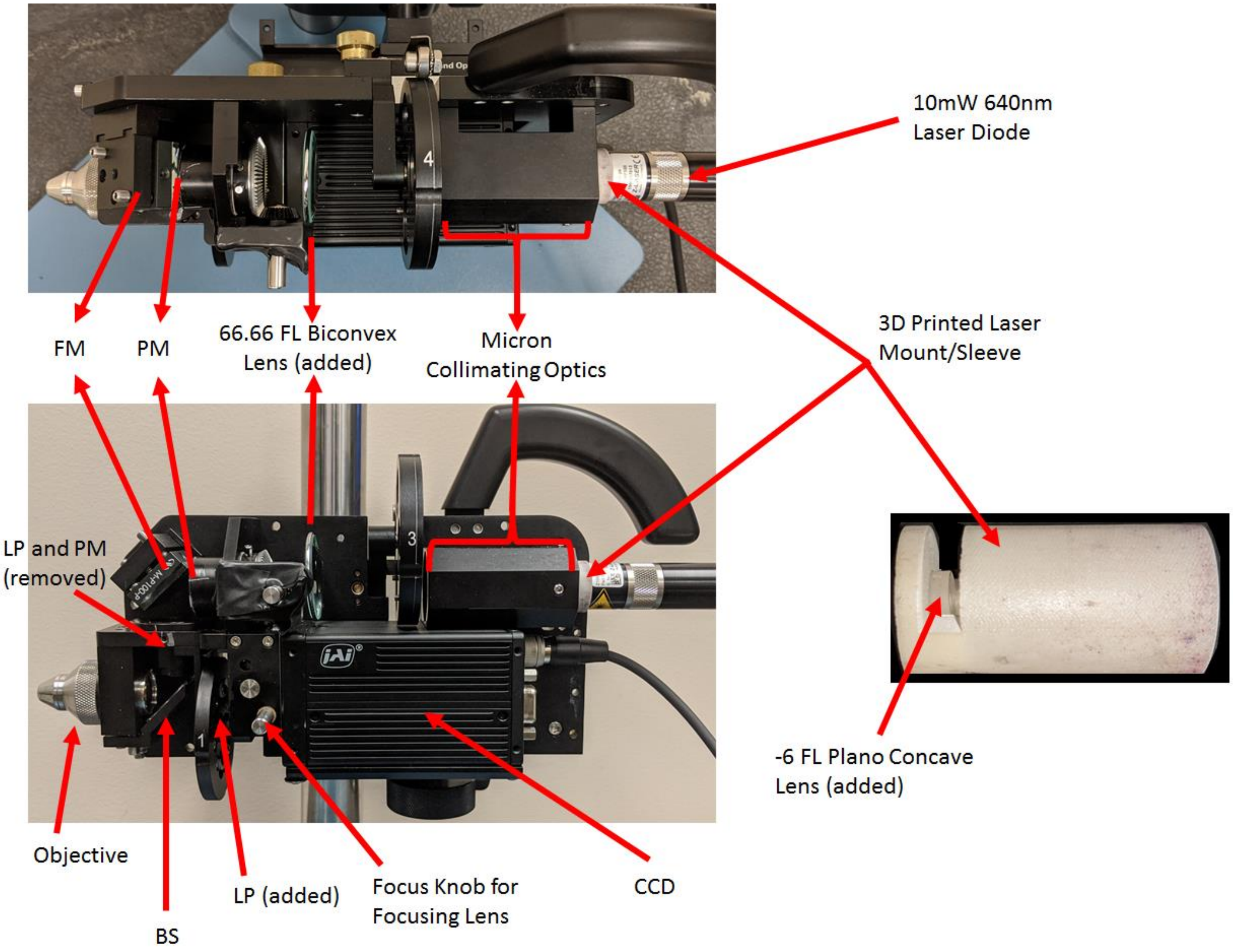

Supplementary Figure S1. Optical Setup of the Modified Micron Fundus Camera for LSCI. A 10mW 640nm laser diode was held in place using a custom 3D printed sleeve which also provided housing for a -6mm focal length (FL) plano-concave lens. A 66.66mm FL biconvex lens was also introduced into the optical path to adjust beam diameter while a pupil mask (PM) and linear polarizer (LP) were removed. A separate LP was introduced between the sample and the charge-coupled device (CCD). Other elements include a fold mirror (FM) and a beam splitter (BS).
